# Childhood Obesity and Craniofacial Growth: A Cross-Sectional Orthodontic Cephalometric Study

**DOI:** 10.3390/medicina62050884

**Published:** 2026-05-05

**Authors:** Sorana Maria Bucur, Dorin Ioan Cocoș, Cristian Doru Olteanu, Mioara Decusară, Mariana Păcurar, Eugen Silviu Bud

**Affiliations:** 1 Department of Dentistry, Faculty of Medicine, Dimitrie Cantemir University of Târgu Mureș, 3-5 Bodoni Sandor Street, 540545 Târgu Mureș, Romania; bucursoranamaria@gmail.com; 2 Faculty of Dental Medicine, “Dunărea de Jos” University, 800008 Galați, Romania; cdorin1123@gmail.com; 3 Department of Orthodontics, School of Dental Medicine, University of Medicine and Pharmacy Iuliu Hatieganu, Str. Avram Iancu 31, 400083 Cluj-Napoca, Romania; 4 Department of Dentistry, Faculty of Medicine and Pharmacy, “Dunărea de Jos” University of Galați, 47 Domnească Str., 800008 Galați, Romania; 5 Department of Orthodontics, Faculty of Dentistry, George Emil Palade University of Medicine, Pharmacy, Science, and Technology of Târgu Mureș, 38 Gheorghe Marinescu Street, 540142 Târgu Mureș, Romaniaeugen.bud@umfst.ro (E.S.B.)

**Keywords:** orthodontics, childhood obesity, body mass index, cephalometry, craniofacial growth, mandibular development, skeletal maturation

## Abstract

*Background and Objectives*: Childhood obesity is a major global health concern and is increasingly recognized as a factor influencing skeletal development. Emerging evidence suggests that excess adiposity may alter craniofacial growth patterns, with potential implications for orthodontic diagnosis and treatment planning. However, the extent to which obesity affects craniofacial morphology in growing individuals remains incompletely understood. This study aims to evaluate the association between body mass index (BMI) and craniofacial morphology in children and adolescents using selected sagittal and linear parameters, and to assess the independent effects of age and sex. *Materials and Methods*: This cross-sectional orthodontic study included 130 subjects aged ≤ 19 years. Anthropometric measurements were recorded, and BMI was used to classify participants as normal weight, overweight, or obese. Standardized lateral cephalometric radiographs were analyzed using skeletal and soft-tissue parameters. Statistical analyses included normality testing, one-way ANOVA with post hoc comparisons, and multivariate modeling. *Results:* Obesity was significantly associated with increased sagittal skeletal dimensions. Mandibular body length, mandibular unit length, SNB angle, and maxillary unit length demonstrated progressive increases across BMI categories (*p* < 0.05). In contrast, vertical growth parameters showed no significant differences. Soft-tissue analysis revealed reduced facial convexity and lower facial height ratios in obese subjects. Age was strongly associated with increases in linear jaw dimensions, whereas sex differences were limited primarily to skeletal size rather than morphological relationships. *Conclusions:* Childhood obesity is associated with enhanced sagittal craniofacial growth, particularly involving mandibular structures, while vertical skeletal patterns remain largely unaffected. These findings highlight the importance of incorporating BMI assessment into orthodontic evaluation and suggest that obesity may influence growth timing, facial morphology, and airway-related risk factors.

## 1. Introduction

Childhood obesity represents a major global public health challenge, with its prevalence increasing substantially over the past several decades [1]. Data from the National Health and Nutrition Examination Survey (NHANES) indicate that prevalence rose from approximately 5% in the 1960s to 10–12% during 1988–1994, reaching nearly 16% by the early 2000s [2]. This trend is accompanied by marked ethnic and socioeconomic disparities. For example, a large survey among primary school children in New York City reported that approximately 41% were overweight and 22% were obese, with higher rates observed among Hispanic and African American populations compared to Caucasian and Asian groups [3].

The etiology of childhood obesity is complex and multifactorial. Genetic predisposition, particularly variations affecting leptin metabolism and appetite regulation, plays a significant role [4,5]. However, environmental and behavioral factors remain key contributors [6]. Sedentary lifestyles, especially prolonged screen exposure, reduce physical activity while simultaneously increasing exposure to advertising for energy-dense foods, thereby promoting excessive caloric intake [2]. Dietary patterns have also shifted toward increased consumption of refined carbohydrates, sugar-sweetened beverages, and high-glycemic foods, contributing to metabolic imbalance and fat accumulation. Psychosocial determinants such as chronic stress, family instability, depression, and low socioeconomic status further increase obesity risk, while parental obesity strongly predicts childhood obesity [2,3].

Childhood obesity is associated with numerous systemic and psychosocial complications. Cardiometabolic consequences include hypertension, dyslipidemia, insulin resistance, and type II diabetes, with approximately 80–85% of pediatric diabetes cases occurring in overweight or obese children [4,5]. Other associated conditions include polycystic ovarian syndrome, fatty liver disease, gallstones, orthopedic abnormalities such as Blount’s disease, asthma, and obstructive sleep apnea related to airway narrowing and craniofacial anatomical factors [6,7]. Beyond physical morbidity, obese children frequently experience reduced self-esteem, social stigmatization, and long-term socioeconomic disadvantages [8].

In recent years, growing attention has focused on the potential relationship between obesity and craniofacial development. Orthodontic research suggests that obesity is associated with measurable differences in craniofacial morphology that may influence airway dimensions and susceptibility to obstructive sleep apnea [9]. Cephalometric studies report that obese individuals often exhibit longer cranial bases, reduced ANB angles, and more anterior mandibular positioning compared with normal-weight peers, indicating a tendency toward altered sagittal skeletal relationships [9,10]. Three-dimensional soft-tissue analyses further demonstrate increased facial widths, larger mandibular dimensions, and deeper facial profiles in obese adolescents, with similar tendencies observed even in mildly overweight individuals [10].

Additional evidence indicates that obesity may be associated with craniofacial development through endocrine pathways, including alterations in growth hormone activity. Cephalometric investigations have reported increased linear measurements of the mandible, maxilla, and anterior cranial base in obese subjects, with the most pronounced differences involving mandibular length [9,10,11]. These findings support the concept that obese children may exhibit differences in skeletal growth timing and maturation patterns during development [9,10,11].

Beyond systemic health implications, childhood obesity has important relevance for orthodontic diagnosis and treatment planning [9,10,11,12]. Because orthodontic patients are typically evaluated during active growth periods, variations in skeletal maturation can substantially influence diagnostic interpretation, treatment timing, and therapeutic outcomes [13]. Obesity-related differences in craniofacial growth may affect sagittal jaw relationships, facial profile harmony, and airway morphology, thereby increasing susceptibility to sleep-disordered breathing [6,7,14].

Despite growing interest in this field, the association between excess body weight and craniofacial morphology remains incompletely understood. Existing studies are often limited by small sample sizes, methodological heterogeneity, and inconsistent evaluation of both skeletal and soft-tissue structures [9]. These limitations highlight the need for integrated investigations combining anthropometric assessment with cephalometric analysis to clarify the extent to which obesity is associated with craniofacial growth patterns [15].

Therefore, this study aimed to evaluate the association between body mass index and craniofacial morphology using selected cephalometric parameters, while also assessing the independent effects of age and sex [11,12]. In addition, the study adopts an integrative approach by analyzing BMI, sex, and age-based developmental groups within a multivariate framework, enabling differentiation between general growth effects and obesity-related craniofacial variation.

## 2. Materials and Methods

### 2.1. Study Design and Ethical Approval

This cross-sectional observational study was conducted in accordance with the Declaration of Helsinki and approved by the Institutional Ethics Committee of Dimitrie Cantemir University (Decision No. 18/18 March 2024). Written informed consent was obtained from all participants and their parents or legal guardians before inclusion.

### 2.2. Study Population and Sample Selection

The study sample consisted of 130 consecutively recruited participants aged between 4 and 19 years who presented for orthodontic evaluation at the Department of Orthodontics, Dimitrie Cantemir University, Târgu Mureș, Romania. Recruitment was conducted over six months between April 2024 and September 2024. All measurements and data collection were performed within this period.

Inclusion criteria were:

- Age ≤ 19 years at the time of examination;
- Absence of craniofacial syndromes or congenital anomalies;
- No history of previous orthodontic treatment;
- Availability of high-quality standardized lateral cephalometric radiographs.

Exclusion criteria included:

- Systemic conditions known to affect growth or bone metabolism;
- History of craniofacial trauma or surgery;
- Radiographs with positioning errors or insufficient diagnostic quality.

Because participants were recruited from an orthodontic population, the sample represents individuals seeking orthodontic care rather than the general pediatric population. This characteristic of the sample should be considered when interpreting craniofacial measurements, as orthodontic patients may present with deviations from normative craniofacial values.

### 2.3. Anthropometric Assessment and BMI Classification

Body weight was measured to the nearest 0.1 kg using a calibrated digital scale, and height was measured to the nearest 0.1 cm using a stadiometer. Body mass index (BMI) was calculated as weight in kilograms divided by height in meters squared (kg/m^2^).

Participants were categorized according to World Health Organization (WHO) BMI-for-age percentile standards:

- Normal weight: BMI between the 5th and 85th percentile.
- Overweight: BMI between the 85th and 95th percentile.
- Obesity: BMI ≥ 95th percentile.

BMI percentiles were calculated using the WHO AnthroPlus software [16]. BMI percentiles were calculated using the WHO AnthroPlus software (version 1.0.4), based on the WHO 2007 growth reference.

### 2.4. Cephalometric Imaging and Analysis

Standardized lateral cephalometric radiographs were obtained using the same digital cephalometric unit under identical exposure parameters. All images were acquired with subjects positioned in natural head posture, teeth in centric occlusion, and lips at rest.

Cephalometric tracing and measurements were performed using Dolphin Imaging software (Version 11.95, Dolphin Imaging & Management Solutions, Chatsworth, CA, USA) by a single calibrated examiner.

The following skeletal and soft-tissue variables were analyzed:

Sagittal skeletal parameters

- SNA angle (°).
- SNB angle (°).
- ANB angle (°).

Linear skeletal measurements

- Maxillary unit length (mm).
- Mandibular unit length (mm).
- Mandibular body length (mm).

Soft-tissue parameters

- Facial convexity angle (°).
- Lower-to-total facial height ratio (%).

### 2.5. Measurement Reliability

To assess intra-observer reliability, 20 randomly selected radiographs were retraced after a two-week interval. Intraclass correlation coefficients (ICCs) ranged from 0.92 to 0.97, indicating excellent measurement reproducibility.

### 2.6. Age Stratification and Growth Phase Classification

Participants were stratified into four groups based on chronological age:

- 4.0–10.9 years.
- 11.0–12.9 years.
- 13.0–14.9 years.
- 15.0–19.9 years.

Stratification was performed exclusively using chronological age. These intervals were selected to reflect clinically relevant developmental periods commonly used in orthodontic research.

Although cervical vertebral maturation (CVM) stages are frequently used to assess skeletal maturity [17,18], CVM was not directly evaluated in this study. Therefore, age groups should not be interpreted as exact indicators of individual skeletal maturation.

The use of chronological age as a proxy for growth phase may introduce misclassification, particularly in populations where growth timing may differ, such as in children with obesity.

**Figure 1:**
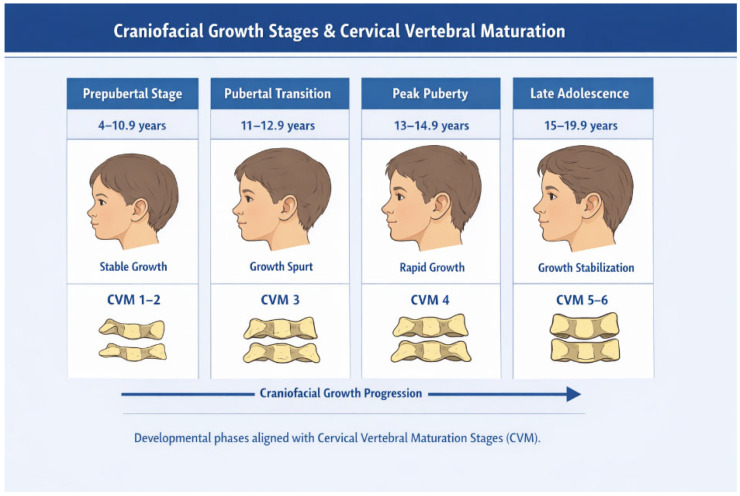
Schematic representation of age-based grouping and its correspondence to cervical vertebral maturation (CVM) stages. The diagram illustrates conceptual alignment rather than direct CVM assessment.

Figure 1 provides a schematic illustration of age-based grouping and should be interpreted as a conceptual representation rather than a direct assessment of skeletal maturation.

### 2.7. Statistical Analysis

Statistical analysis was performed using SPSS Statistics for Windows, Version 26.0 (IBM Corp., Armonk, NY, USA).

The analytical workflow included:

1. Normality assessment using the Shapiro–Wilk test.
2. Descriptive statistics reported as mean ± standard deviation.
3. One-way ANOVA to evaluate differences among BMI groups.
4. Tukey post hoc testing for pairwise comparisons.
5. Independent *t*-tests to assess sex differences.
6. Two-way ANOVA to examine independent effects of age and BMI.
7. Multivariate linear regression analysis to determine independent predictors of craniofacial parameters after adjustment for age and sex. Ninety-five percent confidence intervals were calculated for all regression coefficients.

Effect sizes were evaluated using partial eta squared (η^2^) for ANOVA models.

Statistical significance was set at: *p* < 0.05.

### 2.8. Sample Size Considerations

Based on post hoc power analysis, the sample size provided >80% statistical power to detect moderate effect sizes at α = 0.05.

### 2.9. Potential Sources of Bias

Several potential sources of bias were considered in the present study. First, selection bias may be present, as participants were recruited from an orthodontic population seeking treatment, which may limit generalizability to the general pediatric population and may be associated with pre-existing craniofacial deviations.

Second, the cross-sectional design limits the ability to assess temporal relationships or causality between obesity and craniofacial growth.

Third, residual confounding may persist due to unmeasured variables such as pubertal status, endocrine factors, or respiratory patterns.

Finally, BMI was used as a surrogate marker of adiposity and does not account for body fat distribution or metabolic differences.

## 3. Results

### 3.1. Sample Characteristics

The study included 130 participants, of whom 73% were classified as normal weight, 14% overweight, and 13% obese. Females comprised a slightly larger proportion of the sample. The largest age group corresponded to early-to-mid adolescence (Table 1).

### 3.2. Data Distribution

All cephalometric variables demonstrated normal distribution according to the Shapiro–Wilk test, allowing the use of parametric statistical methods (Table 2).

### 3.3. Craniofacial Differences According to BMI

Significant associations were observed between BMI and sagittal craniofacial dimensions (Table 3). Mandibular unit length, mandibular body length, SNB angle, and maxillary unit length progressively increased across BMI categories (*p* < 0.05).

In contrast, vertical skeletal parameters did not differ significantly among BMI groups.

Soft-tissue analysis showed reduced facial convexity and lower facial height ratios in obese subjects (Table 3).

### 3.4. Sex-Related Differences

Male participants exhibited significantly larger linear skeletal dimensions, including anterior cranial base length, maxillary unit length, and mandibular unit length (*p* < 0.01). However, no significant sex differences were observed in angular skeletal relationships or soft-tissue parameters.

### 3.5. Age-Related Craniofacial Changes

Age was strongly associated with increases in maxillary and mandibular linear dimensions. The most pronounced growth occurred between the prepubertal and early pubertal groups, corresponding to the onset of accelerated skeletal development.

Smaller increments were observed during later adolescence, indicating stabilization of craniofacial growth as skeletal maturity approached.

Overall, BMI was primarily associated with sagittal craniofacial development, particularly mandibular growth and positioning, whereas vertical relationships remained largely unchanged. Age mainly impacts linear skeletal growth, and sexual dimorphism is predominantly related to skeletal size rather than morphology.

### 3.6. Age–BMI Interaction Results

Two-way ANOVA analysis demonstrated significant independent effects of both age and BMI on craniofacial morphology (Table 4). Age exerted a strong influence on linear skeletal parameters, with progressive increases observed in maxillary and mandibular dimensions across developmental stages. The most pronounced growth occurred between the prepubertal and early pubertal groups, followed by smaller increments and near stabilization in late adolescence.

BMI showed a significant independent association primarily with sagittal skeletal relationships. Higher BMI values were correlated with increased SNB angle, greater mandibular body and unit lengths, and reduced facial convexity, indicating a trend toward increased mandibular prognathism in overweight and obese subjects. Vertical proportional relationships remained relatively stable, although facial height ratio demonstrated modest reductions in older and higher-BMI groups.

No significant interaction effect between age and BMI was identified, suggesting that obesity primarily amplifies normal growth patterns rather than altering their developmental timing.

A significant difference was observed among BMI groups for mandibular length (F = 8.45, *p* < 0.001, η^2^ = 0.12), indicating a moderate effect size.

### 3.7. Multivariate Regression Analysis

Multivariate regression analysis confirmed that BMI independently predicted several craniofacial parameters after adjustment for age and sex.

Higher BMI was significantly associated with increased mandibular prognathism, reflected by greater SNB angle and mandibular unit length, as well as reduced ANB angle and facial convexity.

Age remained the strongest determinant of linear skeletal growth, while sex showed a moderate influence primarily related to skeletal size. Table 5 summarizes the regression analysis of factors influencing craniofacial parameters.

**Table 5:** Multivariate linear regression analysis of factors influencing craniofacial parameters.

Dependent Variable	Predictor	β Coefficient	Standard Error	*p* Value
SNB (°)	BMI	0.18	0.05	<0.001
	Age	0.22	0.04	<0.001
	Sex (Male)	0.09	0.03	0.014
Mandibular unit length (mm)	BMI	0.36	0.07	<0.001
	Age	0.48	0.06	<0.001
	Sex (Male)	0.21	0.05	0.002
ANB (°)	BMI	−0.14	0.04	0.001
	Age	−0.09	0.03	0.019
	Sex (Male)	−0.05	0.02	0.087
Facial convexity angle (°)	BMI	−0.31	0.06	<0.001
	Age	−0.28	0.05	<0.001
	Sex (Male)	−0.07	0.03	0.041

All regression models demonstrated good explanatory power, with adjusted R^2^ values ranging from 0.41 to 0.63. Regression coefficients are presented with 95% confidence intervals.

## 4. Discussion

The present study demonstrates that childhood obesity is associated with measurable differences in craniofacial morphology, primarily involving sagittal skeletal dimensions [9,12]. Unlike previous studies that evaluated isolated associations, the present analysis integrates BMI, sex, and growth phase stratification, enabling a more comprehensive understanding of obesity-related craniofacial variation [9,15]. The findings demonstrate a consistent pattern of increased mandibular and maxillary linear measurements in overweight and obese subjects, accompanied by progressive increases in SNB angle and reductions in soft-tissue convexity [10]. These findings indicate a consistent shift toward increased mandibular prominence and altered sagittal relationships in individuals with elevated BMI [10,12,19]. Importantly, vertical skeletal parameters remained largely unchanged, indicating that obesity appears to be selectively associated with anteroposterior craniofacial growth rather than vertical facial development. These observations differ from some previous reports suggesting proportionally increased anteroposterior and vertical craniofacial dimensions in overweight children and adolescents [20].

Obesity-related craniofacial differences were most evident during the pubertal growth phase, suggesting a possible interaction between metabolic status and periods of accelerated skeletal maturation [9,21].

The enlargement of mandibular dimensions represented the most pronounced morphological change identified in this investigation. Mandibular unit length showed the strongest association with BMI, displaying a progressive increase across weight categories. This pattern supports the hypothesis that increased adiposity may be associated with enhanced skeletal growth activity during critical developmental periods [22]. The mandibular condyle, as a major site of endochondral growth, is responsive to systemic metabolic and hormonal influences, and increased growth activity in obese individuals may reflect altered endocrine regulation [23]. Previous studies suggest that hyperinsulinemia and elevated insulin-like growth factor-1 levels, commonly observed in obese children, have been proposed to stimulate chondrocyte proliferation and matrix deposition within condylar cartilage, thereby contributing to increased mandibular growth [24,25]. In addition, leptin and other adipokines secreted by adipose tissue may further modulate bone metabolism and growth plate activity, potentially influencing skeletal enlargement in this population [26,27].

The observed increase in maxillary length in obese subjects, although less pronounced than mandibular changes, indicates that obesity may be associated with broader craniofacial skeletal development rather than isolated mandibular growth alone [9,28]. However, the predominance of mandibular differences suggests a differential sensitivity of craniofacial structures to metabolic and hormonal influences, with the mandible appearing particularly responsive to obesity-related growth variations [29]. This observation is consistent with previous cephalometric and three-dimensional imaging studies reporting larger mandibular dimensions and more prognathic skeletal profiles in overweight adolescents [9,10].

In contrast to sagittal findings, vertical craniofacial parameters did not demonstrate significant differences across BMI groups. This suggests that obesity may not substantially modify vertical growth patterns or facial divergence, reinforcing the concept that the primary craniofacial associations of increased adiposity relate to forward skeletal growth rather than vertical rotational changes [9,30]. The relative stability of vertical measurements across BMI categories also supports the view that intrinsic growth direction is largely genetically determined and less susceptible to metabolic influences [31,32].

Soft-tissue analysis revealed a reduction in facial convexity and lower facial height ratios among obese subjects. These changes likely reflect both underlying skeletal differences and increased soft-tissue thickness associated with adipose deposition [33,34]. The reduction in convexity has clinical relevance, as it may mask or exaggerate underlying skeletal discrepancies, potentially complicating orthodontic diagnosis [35]. Furthermore, altered soft-tissue profiles in obese individuals may have implications for airway-related outcomes, although these were not directly assessed in the present study [36]. The interaction between craniofacial morphology and airway patency represents an important interdisciplinary consideration in pediatric orthodontic assessment [36,37].

Age stratification on chronological groups facilitated comparison across developmental periods but does not replace direct assessment of skeletal maturation. By grouping participants according to prepubertal, pubertal transition, peak growth, and late maturation stages, the analysis accounts for known variations in craniofacial growth velocity, particularly mandibular development, thereby reducing age-related confounding and improving the interpretability of BMI-related skeletal associations [38,39].

Sex-related differences observed in this study were limited to linear skeletal dimensions, with males demonstrating larger cranial base and jaw lengths compared to females. These findings are consistent with established patterns of sexual dimorphism in craniofacial growth and reflect overall differences in skeletal size rather than morphological pattern [40]. The absence of significant differences in angular and soft-tissue parameters suggests that obesity-related craniofacial variations occur similarly in both sexes [9,40].

Age-related analysis revealed expected progressive increases in maxillary and mandibular lengths throughout childhood and adolescence, with the most pronounced growth occurring during early developmental stages [17,18]. The attenuation of differences in late adolescence suggests that skeletal maturation eventually reduces variability in craniofacial dimensions, supporting previous observations that obesity may be associated with earlier attainment of peak growth velocity without necessarily altering final adult morphology [10,29].

From a clinical perspective, these findings underscore the importance of incorporating anthropometric assessment into orthodontic diagnostic protocols [41]. Failure to consider BMI may lead to misinterpretation of skeletal relationships, particularly in cases presenting with apparent mandibular prognathism [19,23,25,28]. Moreover, differences in growth patterns among obese patients may influence the timing and effectiveness of orthopedic treatment interventions, highlighting the need for individualized treatment planning.

The findings of this study should be interpreted as confirmatory yet integrative, reinforcing previously reported associations while improving methodological clarity through multivariate adjustment and biologically based growth phase stratification.

Despite its strengths, this study has several limitations. The use of an orthodontic patient sample introduces potential selection bias, as individuals seeking treatment may present with a higher prevalence of craniofacial discrepancies, including malocclusions and skeletal imbalances. This may influence the observed associations between BMI and craniofacial morphology and limit the generalizability of the findings to the broader pediatric population.

Additionally, the cross-sectional design precludes causal inference and does not allow evaluation of longitudinal growth trajectories. The use of chronological age as a proxy for growth phase, without direct assessment of skeletal maturation (e.g., CVM), may introduce misclassification, particularly in obese children in whom accelerated maturation has been reported. Subgroup analyses involved relatively small sample sizes in the overweight and obese groups, which may limit statistical precision and the robustness of subgroup estimates.

Furthermore, BMI does not reflect body fat distribution or metabolic status. Unmeasured confounders such as pubertal stage, endocrine factors, and respiratory patterns may also have influenced craniofacial development [27,32].

Future longitudinal studies incorporating hormonal assessment and advanced three-dimensional imaging are warranted to further elucidate the biological mechanisms underlying these associations. Unmeasured confounders such as pubertal status, endocrine variations, and breathing patterns may have influenced craniofacial morphology, and the orthodontic sample limits generalizability to the broader pediatric population.

## 5. Conclusions

This cross-sectional study demonstrates that childhood obesity is associated with alterations in craniofacial morphology, predominantly affecting sagittal skeletal relationships. Higher BMI was linked to increased mandibular and maxillary dimensions, greater SNB angle, and reduced facial convexity, indicating a trend toward enhanced mandibular prominence, while vertical skeletal patterns remained largely unaffected.

Age was the principal determinant of overall skeletal growth, whereas sex differences were primarily related to size. Importantly, the associations between BMI and craniofacial parameters remained significant after adjustment for age and sex, supporting an independent relationship between adiposity and craniofacial development.

Clinically, these findings underscore the importance of incorporating anthropometric assessment into orthodontic evaluation. However, given the cross-sectional design, the use of chronological age as a proxy for maturation, and the orthodontic nature of the sample, the results should be interpreted with caution. Further longitudinal studies incorporating direct measures of skeletal maturation are warranted.

## Figures and Tables

**Table 1:** Distribution of participants.

Variable	Category	n
BMI	Normal weight	95
	Overweight	18
	Obese	17
Sex	Male	53
	Female	77
Age group	4–10.9 years	26
	11–12.9 years	39
	13–14.9 years	42
	15–19.9 years	23

Most participants were of normal weight (73%), with a slightly higher female representation. The largest proportion belonged to early-to-mid adolescence.

**Table 2:** Shapiro–Wilk normality testing for key cephalometric variables.

Parameter	W Value	*p* Value	Distribution
SNA	0.972	0.118	Normal
SNB	0.968	0.094	Normal
ANB	0.955	0.061	Normal
Maxillary unit length	0.979	0.164	Normal
Mandibular unit length	0.966	0.083	Normal
Mandibular body length	0.971	0.107	Normal
Facial convexity angle	0.963	0.072	Normal
Facial height ratio	0.976	0.149	Normal

All primary variables demonstrated normal distribution, allowing parametric statistical testing.

**Table 3:** Cephalometric parameters by BMI category.

Parameter	Normal Weight	Overweight	Obese	*p* Value
SNA (°)	82.4 ± 3.0	85.0 ± 3.3	84.1 ± 3.1	0.010
SNB (°)	79.2 ± 2.9	81.5 ± 3.0	82.4 ± 3.2	0.003
ANB (°)	3.2 ± 1.8	3.5 ± 1.7	1.7 ± 1.6	0.012
Maxillary unit length (mm)	97.0 ± 4.1	98.6 ± 4.4	100.1 ± 4.3	0.017
Mandibular unit length (mm)	123.0 ± 6.6	126.4 ± 7.0	129.6 ± 6.7	0.002
Mandibular body length (mm)	74.0 ± 5.1	76.5 ± 5.0	78.2 ± 5.3	0.013
Facial convexity angle (°)	15.3 ± 4.1	12.8 ± 3.8	10.1 ± 3.4	0.005
Facial height ratio (%)	95.4 ± 3.6	91.1 ± 4.0	87.2 ± 4.3	0.007

**Table 4:** Influence of age and BMI on key craniofacial parameters.

Parameter	4–10.9 Years	11–12.9 Years	13–14.9 Years	15–19.9 Years	Age Effect (*p*)	BMI Effect (*p*)
SNA (°)	81.2 ± 2.9	82.5 ± 3.1	83.2 ± 3.3	83.6 ± 3.5	0.039	0.010
SNB (°)	77.8 ± 3.1	79.6 ± 3.2	80.9 ± 3.4	81.3 ± 3.6	<0.001	<0.001
ANB (°)	3.4 ± 1.9	2.9 ± 1.8	2.3 ± 1.7	2.1 ± 1.6	0.021	0.012
Maxillary unit length (mm)	93.5 ± 4.6	96.1 ± 4.4	98.0 ± 4.8	99.0 ± 5.1	<0.001	0.017
Mandibular unit length (mm)	118.3 ± 6.2	123.5 ± 6.6	127.3 ± 7.1	129.0 ± 7.4	<0.001	<0.001
Mandibular body length (mm)	70.0 ± 4.2	73.5 ± 4.6	76.1 ± 5.0	76.9 ± 5.2	<0.001	0.013
Facial convexity angle (°)	16.4 ± 4.6	14.2 ± 4.4	11.9 ± 4.2	11.0 ± 4.3	<0.001	0.005
Facial height ratio (%)	96.6 ± 5.2	92.1 ± 4.9	89.3 ± 4.7	87.7 ± 4.5	<0.001	0.007

Values expressed as mean ± SD. Two-way ANOVA tested the independent effects of age and BMI.

## Data Availability

Data is available upon a reasonable request.
